# Inorganic and Hybrid Perovskite Based Laser Devices: A Review

**DOI:** 10.3390/ma12060859

**Published:** 2019-03-14

**Authors:** Minas M. Stylianakis, Temur Maksudov, Apostolos Panagiotopoulos, George Kakavelakis, Konstantinos Petridis

**Affiliations:** 1 Center of Materials Technology and Photonics & Electrical Engineering Department, Technological Educational Institute (TEI) of Crete, 71004 Heraklion, Crete, Greece; stylianakis@staff.teicrete.gr (M.M.S.); maksudov@staff.teicrete.gr (T.M.); appanagioto@staff.teicrete.gr (A.P.); 2 Department of Materials Science and Technology, University of Crete, Vassilika Voutes GR-700 13, 71004 Heraklion, Crete, Greece; 3 Cambridge Graphene Centre, University of Cambridge, 9 JJ Thomson Avenue, Cambridge CB3 0FA, UK; gk415@cam.ac.uk; 4 Department of Electronic Engineering, Technological Educational Institute (TEI) of Crete, 73132 Chania, Crete, Greece

**Keywords:** inorganic perovskites, hybrid perovskites, stimulated emission, laser devices

## Abstract

Inorganic and organic-inorganic (hybrid) perovskite semiconductor materials have attracted worldwide scientific attention and research effort as the new wonder semiconductor material in optoelectronics. Their excellent physical and electronic properties have been exploited to boost the solar cells efficiency beyond 23% and captivate their potential as competitors to the dominant silicon solar cells technology. However, the fundamental principles in Physics, dictate that an excellent direct band gap material for photovoltaic applications must be also an excellent light emitter candidate. This has been realized for the case of perovskite-based light emitting diodes (LEDs) but much less for the case of the respective laser devices. Here, the strides, exclusively in lasing, made since 2014 are presented for the first time. The solution processability, low temperature crystallization, formation of nearly defect free, nanostructures, the long range ambipolar transport, the direct energy band gap, the high spectral emission tunability over the entire visible spectrum and the almost 100% external luminescence efficiency show perovskite semiconductors’ potential to transform the nanophotonics sector. The operational principles, the various adopted material and laser configurations along the future challenges are reviewed and presented in this paper.

## 1. Introduction

Low temperature solution processed materials that exhibit (a) high optical and tuneable absorption and emission; (b) high optical gain; (c) high crystal quality; and (d) the ability to be integrated into various optical resonator configurations, are features very attractive for a primary gain medium on chip laser devices and applications. In the 1990’s organic semiconductors were the leading contenders of solution processed light emitting and laser devices. Their main drawbacks such as low damage thresholds, low mobilities, non-radiative losses in high charge carriers injection, diverted the research community’s interest to other materials such as inorganic colloidal nanocrystals and now chemically synthesized perovskites [1]. Colloidal semiconductor nanocrystals demonstrate some very unique and attractive features for their application in light emitting devices: high absorption cross section, high quantum yield emission at room temperature, capability to tune their bandgap with the size or their chemical constitution. On top, their solution processability allows their integration on several substrates and photonic structures. However, the colloidal nanostructures demonstrate high amplified stimulate emission thresholds (>mJ/cm^2^) mainly due to high Auger non-radiative rates. The solution processed perovskite semiconductors appear as the alternatives to the colloidal semiconductor nanocrystals. This is attributed to their long radiative recombination time and slower Auger effects that lower the lasing threshold in the range of μJ/cm^2^.

Any material that follows calcium titanium oxide (CaTiO_3_, a cubic unit cell where titanium atoms are at the corners, oxygen atoms at the midpoints of the edges and calcium atoms in the centre) crystal structure—known as perovskite structure (ABX_3_)—is called perovskite material. The CaTiO_3_ mineral was discovered in the Ural Mountains of Russia by Gustav Rose in 1839 and is named after Russian mineralogist Lev Perovski. In general, the perovskite structure consists of a large atomic or molecular cation of type A in the centre of a cube whereas the corners of the cube are occupied by cations B and the faces of the cube are occupied by smaller X anions (negatively charged). Apart of the naturally found perovskite minerals, the chemical structured perovskite materials have attracted the scientific community’s attention and research efforts, due to their outstanding physical and electronic properties in combination with the ease of their processing. Chemical Synthesized organic-inorganic (hybrid) perovskites are the new wonder semiconductor materials in solar cells. Perovskite based solar cells (PSCs) efficiency has skyrocketed beyond 23% within a record period of time [2,3,4,5,6,7]. In this review the chemical synthesized perovskite materials will be referred as perovskite materials.

In 1994, the 1st exhibition of perovskite material-based light emitting device was demonstrated by the IBM research labs [8]. Many of the intriguing electronic and optical properties of halide perovskites such as high optical absorption coefficient, direct energy band gap, large oscillator strength, long carrier lifetime and high quantum efficiency are particularly attractive for light emitting devices. What is more, the photoluminescence quantum yield (PLQY, the ratio of the emitted photons to those absorbed) of metal halide perovskites which is the key parameter that works as a figure of merit for high performance photon emitter, has significantly improved, reaching average values of 40% and 90% in bulk thin films [9] and nanoparticles (NPs) [10,11], respectively. Moreover, controlling the compositional constitution of the perovskite semiconductor’s energy band gap, allows the coverage of the full spectrum when using these materials as active layers in light emitting devices. The solution processability of the hybrid or inorganic perovskite materials permits the fabrication of devices in large scale under lower cost, compared to the respective values for the convectional fabrication methods, such as chemical vapor deposition. Lasers are the youngest member of the family of perovskite optoelectronic devices. Perovskite lasers have entered the stage hand in hand with the vision to create low-cost, solution processed laser diodes. To highlight their potential as a gain medium within a laser system, their lower amplified stimulated emission (ASE) carrier density (how easily a material can acquire net gain) threshold by one order of magnitude compared to those of (a) highly crystalline and high temperature grown ZnSe and CdS; and (b) solution processed organic semiconductors, must be mentioned [12]. This figure of merit provides a strong impetus for optoelectronic applications using perovskite lasers. The low threshold values are attributed to the lower number and slower non-radiative pathways (directly linked to the existence of surface and core defects) compared to the other solution processed materials for example, organic semiconductors. Another attractive feature exhibited by perovskite materials, is their functionality to tune their laser emission wavelengths over the entire visible range (390–790 nm) by (a) modulating the halide constitution (controllable stoichiometry) [13]; (b) varying the lead iodide concentration precursor during the perovskite fabrication process [14]; or (c) by varying or combining the cation element (e.g., using methylammonium (MA) or formamidinium (FA) or using both of them) [15]. Lasing in perovskites can be secured using various cavity/optical feedback architectures: (a) Distributed Feedback provided by gratings [16]; (b) Fabry-Perot resonators formed between the facets of the perovskite thin films [17]; (c) Scattering (in random lasing) provided by various grain boundaries [18]; (d) Whispering Galley Mode (WGM) cavities, exploiting total internal reflection [19]; and (e) polaritonic based laser devices [20]. Perovskite lasers have been demonstrated using a broad range of materials’ nanostructures such as microplatelets, nanowires, photonic crystal corrugations nanodots, nanocubes, microdiscs, quantum dots and so forth. All the important results are summarized in tables that contain for each perovskite material all the important figures of merit: Lasing Threshold, Spectral Linewidth, Tunability, Q-factor, Gain coefficient. These tables will help the reader to observe the progress of the field, compare and view the characteristics of all the demonstrated perovskite laser devices.

Laser devices require gain media capable of achieving (a) population inversion; (b) optical gain above losses; this will be achieved through high quality optical feedback that a well-defined resonator will secure; and (c) efficient outcoupling of the generated coherent laser light. Primary evidence of the lasing onset [21,22] include (a) a kink in the received optical output as a function of the optical pumping intensity; (b) a spectral shrinkage of the output signal; (c) a faster time resolved photoluminescent (TRPL) decaying due to the stimulated emission. The hybrid organic-inorganic perovskites combine the solution processability lend by their organic compound and at the same time the high crystallinity due to their inorganic part. However, the existence of the organic part is the main source of these materials’ instability under their exposure into the environmental hazards. This challenge is addressed with the employment of the inorganic perovskites that demonstrate superior stability under lasing operational conditions compared to their hybrid perovskites counterparts. The inorganic and hybrid perovskite are crystallized in temperatures compatible with flexible substrates (below 200 °C), characteristic that makes them compatible with the emerging printing electronics industry [23,24].

**Figure 1:**
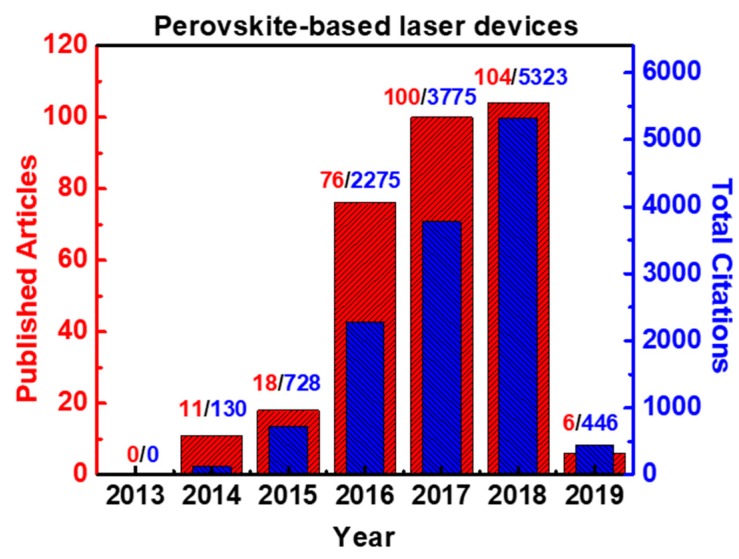
Bar-plot showing the increasing number of published articles (red) and total citations (blue) containing the expressions “Halide Perovskite” and “Laser or Lasing” either in the title or in the abstract during the period 2013 and 2019. Source: Scopus bibliographic database (January 2019).

**Figure 2:**
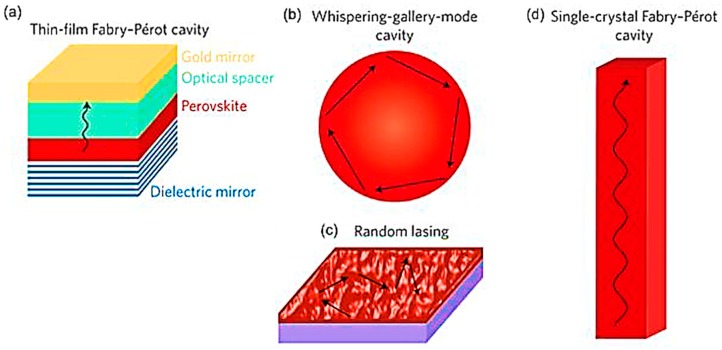
(**a**) The Fabry-Perot Laser Cavity Configuration; (**b**) The Whispering gallery mode cavity; (**c**) The Random Lasing; and (**d**) The single crystal Fabry-Perot Cavity. Reproduced with permission from Sutherland et al. [25], Nature Photonics; published by Macmillan Publishers, 2016.

In this review paper, which is the first attempt to compile all the published results exclusively in lasing devices, we cover the strides made in the field and provide an insight into the major research effort launched since 2014 regarding the application of inorganic and hybrid perovskite materials, as a gain media in laser devices. Previous published excellent review papers have reviewed, to a short extent, lasing devices as a part of other presented photonic devices such as LEDs [1,25]. The advantages the perovskite low dimensional (e.g., nanoplatelets, nanocubes, quantum dots and nanowires) configuration offer, like the formation of natural resonant cavities and their excellent crystal quality are critical parameters for the advancements of nanophotonics. The significantly less done research work in the field of perovskite-based laser devices (compared with the publication explosion in the field of solar cells) was a surprise to us based on the excellent optoelectronic properties of this material and the impact of photonics to the society and Industry world (Figure 1). At the same time, this lag of engagement creates an opportunity window for more research to be implemented in this very promising field. This paper attempts to disseminate and highlight perovskite semiconductors’ high potential as laser gain media through the challenges that field sets. The paper consists of three sections: (a) Hybrid Perovskite based laser systems; (b) Inorganic Perovskite based laser systems; and (c) Challenges and Proposed solutions to advance this very dynamic and with a high impact research topic. Within the first two sections there are three subsections that present and comment the research has been done as a function of the laser cavity configuration selected: (a) Fabry-Perot Cavities; (b) Whispering Mode Cavity; and (c) Random Lasing (see Figure 2). In the third section, solutions of the current challenges this field faces are proposed.

## 2. Literature Review Section

### 2.1. Hybrid Inorganic-Organic Perovskite Laser Devices

#### 2.1.1. Fabry-Perot Laser Cavity Architecture

The Fabry-Perot (FP) Laser Cavity configuration is the most common selected cavity resonator among different architectures the various perovskite-based lasers have adopted. FP laser cavity is formed between at least two reflective surfaces in between the active medium (perovskite semiconductor) is placed. The reflective surfaces are (a) mirrors, for example, gold mirrors; (b) the end facets of a nanowire configuration; or (c) a mirror and a grating in case of Distributed Bragg Reflector Systems (DBR); or (d) a mirror and a photonic crystal. The FP cavity based perovskite lasers offer a lot of control in the spectral and power output of the laser. More specifically offers (a) tunability of their output frequency through tuning of the laser cavity length (which is an extra degree of freedom apart of the tunability demonstrated through the composition of the hybrid perovskite semiconductor); (b) high Q-factors due to the efficient optical trapping of the coherent laser radiation between the two reflectors; (c) low lasing threshold values; (d) high spectral purity; and (e) provides a single directional laser output. In the case of the DFB FP perovskite lasers the optical feedback offers also substantially better operational stability and single mode operation. Q-values up to 3600, threshold as low to 220 nJ/cm^2^ have been exhibited. On the other hand compared to the other two laser cavity configurations that are based on total internal reflection (Whispering Gallery Mode Cavity, WGM) and on random scattering between the grains of the perovskite crystals, is more complicated since demands the engineering of the two reflectors.

**Figure 3:**
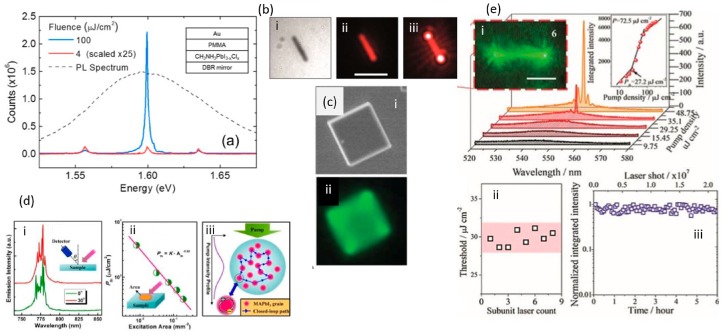
(**a**) Lasing from a Fabry-Perot type Perovskite Laser. Reproduced with the Permission from Deschler et al., J. Phys. Chem. Lett. [26]; published by ACS Publications. (**b**) (**i**): Lasing from nanowire construction; its main emission characteristic from the two edges. (**ii**): Two of the characteristics of the onset of lasing operation, the nonlinear increase of the output intensity and the shrinking of the FWHM of the output above threshold point. Reproduced with the permission from Zhu et al., Nature Materials [27]; published by Macmillan Publishers Limited. (**c**) Whispering Gallery Mode Emission from perovskite microdiscs (**i**) below threshold and (**ii**) above threshold. Reproduced with the permission from Liao et al., Advanced Materials [28]; published by 2015 WILEY-VCH Verlag GmbH & Co. KGaA, Weinheim. (**d**) (**i**) the optical excitation of the samples and the detection of random lasing in different angles. This is one of the main characteristics of lasing due to provided optical feedback from scattering effects. In (**ii**) the variation of the lasing threshold as a function of excitation area on a logarithmic scale. In (**iii**) the principle of the random lasing and the scattering effects between the perovskite grain sizes. Reproduced with the permission of Shi et al. Journal of Materials Chemistry C [29]; published by Royal Society of Chemistry. (**e**) (**i**) Lasing from RPPs 2D perovskite nanowire arrays, the appearance of a threshold point characteristic to lasing; (**ii**) Lasing under pumping at 400 nm with a fluence 80 μJ/cm^2^ and the demonstrating impressive stability these 2D systems demonstrate: lasing for more than six hours. Reproduced with the permission of Zhang et al., Angew. Chem. Int. Ed. [30]; published by 2018 Wiley-VCH Verlag GmbH & Co. KGaA, Weinheim.

Deschler et al. [26] were the first to demonstrate the emission of stimulated light from a vertical cavity laser configuration that enclosed a solution processed methylammonium lead mixed halide film between its two mirrors. The recorded photoluminescent quantum efficiency (PLQE) of the optically pumped films under high pumping densities (>100 mW/cm^2^) was surprisingly high (~70%) showing the existence of very few nonradiative recombination pathways. Lasing operation at room temperature (Figure 3a), for optical pumping energy above 0.2 μJ/pulse (using ns laser systems at 532 nm), was observed; it was reconfirmed through an order of magnitude increase in the slope of the output curve as a function of the pumping power.

**Figure 4:**
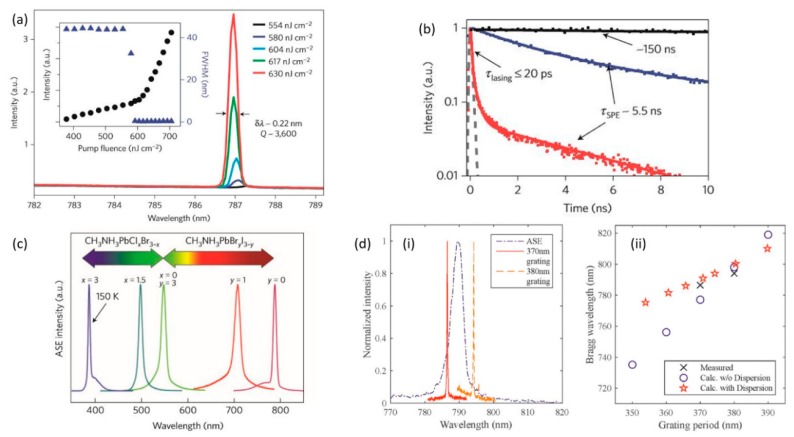
(**a**) Nonlinear increase of the output intensity beyond the laser threshold point; A spectral linewidth shrinking above threshold is also observed as a clear indication of the onset of the laser operation. (**b**) The transition from the spontaneous emission to stimulated emission is depicted on the reduction of the PL signal above threshold as the Time Resolved Photoluminescent measurements indicate. Reproduced with permission from Zhu et al. [27], Nature Materials; published by Nature Publishing Group, 2015. (**c**) Coarse Tunability of the emission laser light achieved through the halide constitution (controllable stoichiometry). Reproduced with the permission from Xing et al. [13], Nature Materials; published by 2014 Macmillan Publishers Limited (**d**) Fine wavelength tuning using different grading periods in a DFB configuration: (**i**) amplified spontaneous emission spectrum and lasing spectra and (**ii**) calculated and measured Bragg wavelengths. Reproduced with permission from Brenner et al. [31], Applied Physics Letters; published by AIP Publishing.

The demonstration of lasing from single crystal halide perovskite nanowires, provided nanolaser systems with strong photon confinement, excellent waveguide properties, high PLQE (~100%) and high Q-cavity factors (~3600). The demonstrated low threshold (~220 nJ/cm^2^) (Figure 3b), was a real breakthrough in the field of semiconductor nanowires (NWs) since it was the main obstacle for their wide use application and the introduction of electrically driven lasers. Zhu et al. [27] work showed that single crystal perovskite NWs (~20 μm length and with hundreds of nm in width) are ideal laser sources for the nanophotonic related Industry (see Figure 4a,b). A MAPbI_3_ excellent crystal quality allowed the NWs two end facets to form a Fabry-Perot (FP) type of resonator, necessary for the building of the stimulated light at room temperature. The remarkable laser operation performance of the MAPbI_3_ NWs was also confirmed from TRPL measurements; the lifetime of the spontaneous emission decreased above threshold from 150 ns to 5.5 ns as a result of stimulated emission (Figure 3b). Single and multimode operation were observed with the distance between the adjacent modes to decrease with the NWs length. The lasing output from such structures was observed to be linearly polarized (degree of polarization (DOP), ~90%) with high polarization purity; the electric field oscillated in the plane (TM) of the NWs.

A step towards the realization of practical hybrid inorganic-organic halide perovskite semiconductor lasers was done by Chen et al. [32]. The authors of this work embedded a solution processed thin film (~130 nm the thickness) of polycrystalline CH_3_NH_3_PbI_3_ between the facets of a two-dimensional photonic crystal in order to realize a photonic crystal (PhC) based microlaser. High degree of single mode operation, temporal and spatial coherence were observed during the lasing operation of the proposed set up. The excellent crystal quality of the solution processed perovskite films secured excellent optical and electrical properties essential for pursuing low threshold laser devices. The practicability of the exhibited system was also confirmed from the pumping source; the authors employed ps laser pulses (lower cost devices than the fs ones) to create the necessary population inversion (optical gain) for lasing. Beyond the threshold (~68 μJ/cm^2^) the lifetime of the spontaneous emission was shortened by 50-fold, from ns to ps range as a clear indication of a gain switch to the lasing regime. The degree of polarization (DOP) was close to 70% for pumping above the lasing threshold point. Tuning the output laser wavelength (for more than 30 nm) under fixed composition, was also achieved by controlling the PhC’s pitch periodicity. The fact that the laser operational conditions induce thermal stress of the perovskite active layers, two to three orders of magnitude higher compared to corresponding values in solar cells, make laser-based devices an excellent platform for photostability measurements.

The fabrication of nanolasers based on the nonlinear properties of the hybrid perovskite semiconductors was realised by Gu et al. [33]. Two photon optically pumped laser devices are attractive since they can be placed within complex environments for example, biological tissues and have a great impact on nonlinear optics; imaging, optical limiters and nanolasers due to the high resolution they can provide. This was the first work that demonstrated the two-photon absorption and lasing emission from microwire (10–30 μm in length) hybrid organic-inorganic perovskite semiconductors (MAPbBr_3_).

Auger recombination mechanism is one of the detrimental factors to achieve low threshold lasing values. This recombination mechanism is extremely strong under high optical pumping rates. Even though the work was performed in the field of perovskite lasers, the strong optical excitation power regime has not been studied in depth. Zhang et al. [34] were the first ones to investigate this pumping regime. Moreover, they suggested that the incorporation of a thin layer of graphene next to the hybrid perovskite nanorods operated as an electron quencher; as a result, the holes and electrons were diffused in different regions. This helped avoid secondary absorption mechanisms that elevated the lasing threshold. The inclusion of a graphene layer on top of the deposition substrate the CH_3_NH_3_PbBr_3_ micro rods were deposited, resulted in the reduction of lasing threshold (reduction by 20%) and the increase by four times of the output intensity under high pumping powers. This lasing operation improvement was attributed to the lowering of the Auger recombination rate due to the electron attractive nature of the graphene layer.

Realization of highly periodic perovskite laser arrays with variable cavity sizes, for large area optical gain applications, was a challenge regarding the demonstration of coherent multi-laser source for optical communication purposes. Liu et al. [35] managed to show direct lithographic patterning of perovskite based optical cavities onto traditional silicon substrates for optoelectronic applications, a task that prior to this work requested complicated lithographic techniques. The authors of this work deposited onto h-BN/SiO_2_ substrates, MAPbI_3_ platelets of various lengths. The insulating h-BN (operated in this work as a buffer layer) was periodically patterned, deposited onto SiO_2_ and continuously the perovskite semiconductor based optical cavities nucleated onto this h-BN/SiO_2_ compound. The MAPbI_3_ platelet-arrays were optically pumped using fs laser pulses at 400 nm. The threshold level was measured at 11 μJ/cm^2^ (emission at 780 nm). TRPL measurements also indicated a fast recombination beyond threshold due to the lasing operation (where the lifetime of spontaneous emission reduced to ~30 ps). Single laser mode and Free Spectral Range (FSR) tunability were attainable through the modulation of the platelets’ size. Moreover, the authors realised a dependence of the achieved lasing thresholds as a function of the cavity length; as the platelet size increased the threshold decreased. This was attributed to the higher Q-factors (better coupling of the spontaneous emission into the stimulated emission) achieved for the longer platelets. On the other hand and again due to FSR increase, single longitudinal mode operation was achieved for a 2 μm cavity. To overcome the high threshold values the short cavities suffered, the authors designed an asymmetrical shaped cavity. In this case the lasing threshold for single mode operation was lowered from 37 μJ/cm^2^ to 12 μJ/cm^2^ (asymmetrical shaped cavity).

One of the challenges the hybrid organic-inorganic perovskite semiconductors face, is that they are susceptible to environmental hazards; this is mainly due to the organic part of this composite materials. In laser-based devices this issue is mainly addressed with the employment of inorganic perovskite materials, mainly Cs based. Brenner et al. [31] motivated by the need of low cost, stable and tuneable laser sources, proposed something different and innovative: the utilization of MAPbI_3_ distributed feedback (DFB) systems as stable and tuneable laser source (see Figure 4d). The authors studied the lasing performance of a perovskite DFB lasers, fabricated in ambient atmosphere, directly on top of low-cost polymer gratings. Stable laser operation (of encapsulated devices) for 15 h under optical pumping (using ns DPSSL at 532 nm, 270 kW/cm^2^) without any obvious change of the emission wavelength (@ 786.5 nm) was observed. This stability was considerably superior to other tuneable active materials, such as organic semiconductors. While the laser threshold value was measured at 120 kW/cm^2^. Tunability of the laser wavelength from 781 to 794 nm was also demonstrated through modification of the grating’s period.

The improvement of the thermal and photo stability of the methylammonium hybrid organic inorganic perovskite materials is a challenge. Fu et al. [36] proposed the use of formamidinium or of an alloy of methylammonium and formamidinium based hybrid halide perovskites (FA/MA composites assisted to fill in the gap of lasing wavelength previously unavailable with MA-based perovskites). Their proposal of solution processed formamidinium lead halide materials that demonstrated superior thermal stability compared to the methylammonium lead halide perovskites (due to better hydrogen bonding between the organic cation and the iodide anions) exhibited laser operational stability for more than 10^8^ pumping 150 fs laser pulses at 402 nm and at room temperature. Single mode laser operation at 800 nm was observed, with the threshold value at 6.2 μJ/cm^2^. Spectral tunability of the output laser wavelength was also demonstrated through cation and anion substitution modifications.

Another type of hybrid halide perovskite NWs was proposed by Yu et al. [37]. The fabricated single crystal hybrid perovskite nanowires were employed as an active layer in plasmonic based laser devices. The latter laser architecture is very attractive and in comparison to Fabry-Perot (FP) nanowire based devices, can generate light below diffraction limit to subwavelength limit. The construction consisted of CH_3_NH_3_PbX_3_ NWs deposited onto a silver film with a 10 nm thick magnesium fluoride (MgF_2_) spacer layer. These systems are of low cost, facile to produce and of high optical gain and are competitors and alternatives to corresponding high cost systems fabricating using VI and III-V inorganic semiconductor NWs. The laser mode of the CH_3_NH_3_PbX_3_ NWs was confined in an ultra-small area, perpendicular to the nanowire axis. The NWs end facets operated as mirrors to form a FP optical resonator, necessary for lasing. The plasmonic CH_3_NH_3_PbX_3_ NW laser output, compared to the photonic respective NW device’s (deposited directly on a quartz surface) output, was characterised by a lot of distinctive characteristics: (a) free of diffraction limit outputs (this is not the case for the photonic devices); and (b) linear polarization across the nanowire axis (while the photonic devices characterised by perpendicular to the optical axis polarization, thin (less than 300 nm) nanowires were required to have distinctive plasmonic devices). The CH_3_NH_3_PbX_3_ NW laser devices were optically pumped employing 120 fs laser pulses at 400 nm with a repetition rate of 1 kHz. Depending on the size (length) of the CH_3_NH_3_PbX_3_ NWs, the threshold varied from 13.5 to 56.3 μJ/cm^2^ at room temperature whereas the wavelength was tuned from 767.76 to 796.75 nm. The threshold pump power increased as the temperature increased. Lasing operation was observed up to temperatures of 43.6 °C.

Trihalide perovskites possess very attractive physical properties that combined with their solution processability, convert them as very promising materials in nanolaser devices. Sarritzu et al. [38] exhibited continuous wave (CW) and quasi CW laser operation using trihalide perovskite-based devices. All the systems were optically pumped using fs laser pulses (130 fs) for the case of CW and ns laser pulses (4 ns or 300 ns) for the quasi CW laser operation. The authors’ main findings included (a) the dependence of the device’s lasing threshold with the square of lattice’s temperature; (b) the thermal equilibrium between the electron—hole plasma with the perovskite lattice when the system was pumped with fs laser pulses; and (c) the existence of a maximum lattice temperature above which there is no light emission due to high threshold values (losses mainly due to non-radiative recombination) when the system was pumped using ns laser pulses. The lattice warming was attributed to (a) Auger non-radiative recombination; and (b) to quantum defect of the excited carriers (ratio between the radiative recombination to the total number of excited carriers). This work highlighted the role and the importance of the deposition substrate not only on the crystallization of the perovskite but also as a heat conveyor.

There is a strong demand in ultra-small laser sources due to the rapid progress of the optical integrated circuits. Up to now, the nanolaser research was based on the optimization of individual devices. However, high impact applications demand an increase in the throughput of nanolaser configurations. This requirement requires the development of nanolaser arrays. The challenges for this technology are numerous. Among them, are: (a) emission in uniform wavelengths; (b) high integration density; and (c) relatively high powers. Sun et al. [39] studied how to build a nanolaser perovskite (CH_3_NH_3_PbBr_3_) based array that emitted high spectral purity output and high output powers using a simple method. The lead halide perovskite semiconductor material was selected due to its high optical gain and high crystalline quality. The optical oscillator in this work, consisted of the lead halide perovskite nanoribbon and the gold grating onto which the perovskite semiconductor has been placed. The refractive indices differences between the grating’s gold teeth and the air slot area around it, provided satisfactory waveguide properties to trap the light in the air slot areas and thus allowed laser action to be observed. The nanolaser arrays secured low threshold due to the excellent waveguiding provided within the air-gaps between the gold made grating teeth. The system was pumped using 100 fs laser pulses at 400 nm. The lasing threshold was measured at 4.2 μJ/cm^2^ at 553 nm with FWHM of around 0.7 nm beyond threshold point. The output was linear polarized (TM—the electric field perpendicular to the plane). The authors highlight that an essential parameter to secure all the desired properties a laser array should have (see above), was the length of the air gap between the grating teeth. There is an optimum value (350–400 nm) that secures the smallest FHWM; an indication of good trapping. The optimum air gap width secured an integration of arrays density of around 1250–1428/mm.

The fabrication of semiconductor nanowires (SNWs) laser arrays is very challenging due to difficulties to directly fabricate these constructions onto silicon substrates. The introduction of cost effective and massive fabrication processes of SNW laser arrays is of high priority for nanophotonic industry. Liu et al. [40] proposed a fabrication technique that can be directly applied onto any substrate. The authors managed to design and fabricate a template onto PDMS (polydimethylsiloxane) over which solution processed MAPbX_3_ nanowire arrays were grown. The spatial confinement the proposed template offered, permitted the full control of the nanowire dimensions (width, length and height) and interwire distance. The nanowires operation principles were governed by the Fabry-Perot cavity principles. These optically pumped (at 400 nm using 150 fs laser pulses) characterized by long lifetime nanowires, corresponded to the emission of 4 × 10^7^ fs laser pulses at 543.1 nm under ambient conditions. The onset of the lasing was accompanied by a substantially increase in the output and a narrowing of the emission spectral width (from 10 nm to 0.9 nm). The threshold value was measured at 18.3 μJ/cm^2^ for PNWs of length of 30 μm. TRPL measurements also demonstrated the effect of the stimulated emission; below threshold the PL signal ceased within 3.6 ns whereas above threshold the PL signal ceased much faster and within 30 ps. As expected and due to higher provided optical gain, the threshold of this architecture decreased as the cavity length got longer. On the other hand, the longer the length of the PNWs the longer its demonstrated operational stability.

Shunemann et al. [41] proposed for the first time an all-solution CH_3_NH_3_PbBr_3_ and CH_3_NH_3_PbI_3_ distributed feedback lasers based on a 3D photonic crystal nanostructure. The exhibited constructions demonstrated very low lasing threshold (1.6 mJ/cm^2^) and long-term operational stability. The proposed fabrication technique is of low cost and does not need the use of expensive and elaborate lithography. The DFB structure ensured the refractive index contrast, between 3D arrays of spheres of air and the perovskite template surrounding these spheres to provide the necessary optical feedback in order to reach lasing.

Cha et al. [42] were motivated by the request to reduce the cost of single mode laser devices based on single-crystalline III-V compound semiconductors. They proposed the use of solution processed halide perovskite alloy system, CH(NH_2_)_2_Pb(I_1−x_Br_x_)_3_. The emission wavelength extracted from the thin film of CH(NH_2_)_2_Pb(I_1−x_Br_x_)_3_ (200 nm thick, coated onto fused quartz substrates) films, could be controlled through the anion composition ratio x (could be tuned from 550 to 820 nm). The alloyed films exhibited a single mode lobed emission at 630 nm (optically pumped by a frequency doubled 532 nm Nd:YAG 400 ps laser, with repetition rate of 1 kHz). The most important information acquired through the PL measurements, was that the alloy was a completely different light emitting semiconductor material than the individual pristine constituents (FAPbBr_3_ and FAPbI_3_); its emission wavelength was between the emission wavelengths of the pristine light emitting semiconductors. The fused quartz substrates onto which the thin film of CH(NH_2_)_2_Pb(I_1−x_Br_x_)_3_ was spin coated (with SEM revealing grain sizes between 100 and 200 nm), were engraved with a grating in order to secure optical feedback. This optical feedback not only reduced the measured lasing threshold (3.5 μJ/cm^2^) but also determined the single mode lasing operation. The selection of the second order diffraction beam permitted the authors to have an additional output beam across the vertical direction of the films’ plane. The composition of the employed perovskite did not only affect the emissive wavelength but also determined the temperature range over which single mode operation appeared. For x = 0 and one, single mode operation demonstrated up to room temperature and up to 200 K for the alloys (x = 0.6). It has been noted that as the temperature elevated, the threshold value followed the same trend. The lasing output from the perovskite alloys was strongly linear polarized, with polarization plane to be parallel to the grating’s lines.

Chen et al. [43] demonstrated a solution processed CH_3_NH_3_PbBr_3_ perovskite microsheet and microwire Fabry-Perot nanolaser devices operating at room temperature. The Fabry-Perot nature of the laser cavity allowed the demonstration of multi-mode and single mode operation by just modifying the optical resonator’s length. The demonstrated systems were optically pumped using 150 fs laser pulses at 325 nm at a repetition rate of 1 kHz. The lasing action was achieved for pump powers beyond 0.251 mW and lasing appeared in the sides of the microsheet. The lasing was accompanied by a sharp increase of the output intensity (transition from the amplified spontaneous emission to stimulated emission) and a shrinkage of the FWHM of the output light: from 25 nm down to 0.41 nm. The emission wavelength was at 554 nm and the calculated quality cavity factor, Q-factor was estimated close to 1352. To lower the threshold level as much as possible and at the same time to secure single longitudinal mode operation, the optimum dimensions of the nanosheet should be calculated (in collaboration of course with the gain bandwidth of CH_3_NH_3_PbBr_3_); the optimum width was estimated as 2.35 μm. Single mode operation was also achieved from CH_3_NH_3_PbBr_3_ nanowires with width of 2.43 μm and length of 380 μm. The respective values of Q-factor, lasing threshold level and output emission was 1924, at 0.357 mW and at 558 nm respectively.

The demonstration of continuous wave (CW) lasing operation by solution processed hybrid perovskite semiconductors is a high priority objective. CW lasing operation is considered the milestone before an electrically pumped diode laser system can be realised. Recent attempts in the case of hybrid perovskites (MAPbI_3_) for CW laser operation has been achieved at 160K. At these temperatures the lifetime of the CW lasing is very short, in the order of few tens of nanoseconds. Jia et al. [44] demonstrated MAPbI_3_ thin films (of thickness of 120 nm) as gain media in a DFB (~120-nm-thick MAPbI_3_ film onto a Λ = 385 nm period grating etched into a thin alumina layer on a high-thermal-conductivity sapphire substrate). CW optically pumped laser device operating at temperatures lower than 160 K, was demonstrated for more than an hour. The threshold level of this optically pumped (at 445 nm from a 920 ns long pulses) systems was determined at 17 kW/cm^2^. The longer lifetime of the CW lasing was attributed to the higher values of population inversions that can be achieved at low temperatures. Moreover, the long lasing in this MAPbI_3_ systems was realised at temperatures where the perovskite crystal has an orthorhombic phase and was transformed to tetragonal when was pumped by high intensity laser pulses. The tetragonal phase inclusions trapped more efficient the carriers and thus achieving higher population inversions and longer-lived CW lasing. When the optical pumping was turned off, the tetragonal phase changed back to orthorhombic and the lasing ceased (at higher temperatures than 160 K).

The integration of efficient light sources for the next generation silicon nitride (Si_3_N_4_) photonic platforms is quite challenging. Technology experts would like to overcome the obstacle of the use of external light sources in silicon nitride optoelectronic platforms; todays’ technology limitations lead to (a) higher coupling losses; and (b) higher required energy per transferred bit. These obstacles are possible to be tackled with the introduction of perovskites nanolasers. These optical devices are (a) possible to be embedded with Si_3_N_4_ waveguide wafers; and (b) do not require complex epitaxial bonding processes (as semiconductors III-V, request) that generate high costs. These credentials are attributed to their solution processability and their low temperature crystallization, which enable them to be easily integrated to existing photonic circuits. The 1st demonstration of an on chip nanolaser device was demonstrated in 2017 by Cegielski et al. [45]. The authors exhibited the integration of solution processed perovskite laser onto a silicon nitride photonic chip. The authors managed to embed methylammonium lead tri-iodide (MAPbI_3_) in a pre-patterned micro-resonator and coupled the laser light into an intergraded photonic waveguide. The integrated laser light was optically pumped, with a threshold value of 19.6 μJ/cm^2^ (optically pumped using 120 fs laser pulses at 645 nm). The authors selected to adopt, for their optical resonator, a racetrack type of cavity. The latter requires much lower lithographic resolution and enables the coupling of laser light into a silicon-nitride waveguide through the emitted evanescent field waves.

Three photon processes in perovskite microlasers were first studied in the work implemented by Gao et al. [46]. The solution processed synthesized hybrid perovskite nanostructures was dominated by microplates and microrods structures. The boundaries and the surfaces of the perovskite nanostructures were very smooth and uniform, a property that assisted the optical feedback required for the lasing operation. The nanostructures were optically pumped using 100 fs laser pulses at 1240 nm. The employed wavelength triggered the three-photon absorption by the perovskite nanostructures. Beyond a lasing threshold point (130 mJ/cm^2^ for the microrods), a non-linear enhancement of the PL signal was observed. Lasing was accompanied by strongly linear TE polarised beam at 547 nm.

Miniaturisation of laser devices is a key concept to lower energy consumption, for faster, smaller and of higher density photon-based sources. The most prominent way to reach this objective, is to adopt the plasmonic based device architecture. The latter can result in devices that break the diffraction limit because they explore the plasmonic effect: the storage of energy in electron oscillations at the metal dielectric interface. Another approach related to the plasmonic configuration, is the generation of oscillating waves in the space between metal nanoparticles and the dielectric (plasmonic waveguide-based spacers) surrounding the nanoparticle. This strategy is more energy advantageous since it is less susceptible to Ohmic losses. The stability, the tunability of the plasmonic waveguide-based spacers is primary defined by the corresponding characteristics of the employed semiconductor. Huang et al. [47] applied hybrid organic-inorganic perovskite as a semiconductor material in a plasmonic waveguide-based spacer (MAPbX_3_/SiO_2_/Au film). The total internal reflection provided in the interface between the perovskite and the air allowed the generation of whispering gallery modes (WGM) in the plane of the perovskite. The high reflectance in the interface between the perovskite & the air, the small oscillating volume and the high optical gain of the perovskite, resulted into a hybrid nanostructure perovskite-SiO_2_-Au nanoscale coherent light source. This miniaturized laser system was pumped using a frequency doubled Ti: Sapphire laser (@400 nm, 100 fs pulse duration and 1000 Hz repetition rate). Below threshold the collected output signal from the hybrid spacer, had a spectral width of 40 nm centred at 770 nm; when the pump power exceeded 59.2 μJ/cm^2^, the emission intensity ‘jumped’ to higher values and the emission spectrum was dominated by the sharp peaks. It was found that when the spacer operated at the plasmonic mode (for perovskite thicknesses below 50 nm), the threshold increased with the thickness of the device whereas when the nanolaser operated under the photonic laser mode the threshold kept much lower at larger thicknesses. The employed perovskite materials, in comparison with previous employed inorganic semiconductors CdS and GaN, can be tuned through halide elements stoichiometry; the detected output coherent light emission wavelengths could be tuned from 550 (CH_3_NH_3_PbBr_3_) to 770 (CH_3_NH_3_PbI_3_) nm. The option to construct a plasmonic nanolaser with an arbitrary cavity shape, as this was determined by the shape of the bottom Au pattern, was explored by the authors. This resulted in much lower laser threshold pump intensities as low as 30 μJ/cm^2^ and control of the resonance frequencies as a function of the shape and the size of the bottom Au patterns. The authors also demonstrated that the resonances within hybrid plasmonic nanolaser can be precisely controlled by the shape and size of bottom Au patterns instead of the top semiconductors. As a result, by patterning the substrate into Au disks and Au strips, they have experimentally realized the circular hybrid plasmonic nanolaser and the uniform plasmonic nanolaser array.

One of the key challenges to introduce electrical pumped perovskite-based lasers is to lower the lasing threshold point as much as possible. Optical feedback using diffraction gratings or distributed feedback (DFB) resonators is a very successful strategy that has already proved its success in inorganic semiconductor laser systems. Pourdavoud et al. [48] have applied the DFB approach on methylammonium lead bromide (MAPbBr_3_) thin films-based laser systems. More particular, a linear photonic grating was directly imprinted onto the MAPbBr_3_ active layer films using the thermal nanoprinting technique (@ 100 °C). The thermal nanoprinting technique also overcame internal limitations the hybrid perovskite demonstrates for example, low intrinsic stability and their susceptibility to a few commonly used solvents, that other strategies have experienced in order to realise high Q-cavities such as wet-chemical lithography. As a result of the nanoprinting linear Bragg grating (300 nm the periodicity of the grading, with a depth of 100 nm) onto the MAPbBr_3_ film, a high Q resonator resulted. The thermally printed grating also resulted in the smothering of the MAPbBr_3_ film surface to roughness below 0.6 nm (from the 46 nm the as cast spin coating MAPbBr_3_ films exhibited). The smooth surface afforded a significant decrease of the threshold of the stimulated emission; In optically pumped (at 532 nm using a 0.3 ns pulsed laser source) DFB laser structures, thresholds as low as 3.4 μJ/cm^2^ have been reached. The stimulated emission output could be tuned in the spectral region between 543.3 to 557.4 nm. The appearance of the lasing was accompanied with the shrinking of the FWHM of the output to 0.14 nm (compared to the 2.5 nm Amplified Spontaneous Emission has shown). The excellent material quality as a result also of the nanoprinting technique was confirmed through SEM images that showed perovskite grain sizes of the order of 10 μm.

Wang et al. [49] provided a deep study of the modulation of laser threshold and emitting laser wavelength as a function of temperature. Such a study was missing before the publication of this work. More particular, in this paper a MAPbI_3_ semiconductor is embedded in a vertical Fabry-Perot (FP) microcavity (consisting of a top metallic mirror and a bottom dielectric distributed Bragg reflector (DBR) with a Q value of 312). This microcavity (PM) system was pumped by a continuous wave (CW) at 633 nm and the lasing threshold measured at 12.9 μJ/cm^2^. Beyond laser threshold, a FWHM of 0.76 nm at 782 nm was measured (@ 25 K). Due to the suppression of the free exciton irradiative recombination as the temperature decreased (from 75K to 25K), the achieved lasing threshold was lowered (as a result of the weaker exciton—phonon interaction and defect scattering), a spectral red shift (as a result of the temperature dependent orthorhombic phase) was observed (750–760 nm) and the spectral coherence was increased. It was registered that beyond 55 K, the lasing operation ceased and the stimulated emission was replaced by the spontaneous emission radiation (broader spectral emissions of lower intensity).

Miniature size laser sources are very attractive for on chip photonic integration technologies. NW configuration laser sources satisfy all the set standards, owing to its ultra-compact size, provided optical confinement and highly localised coherent output. Until recently the only demonstrated hybrid perovskite laser systems were demonstrated using 3D perovskite NWs that mainly suffer from atmospheric moisture and halide ion migration. The solution to these challenges is offered by the two-dimensional (2D) organic-inorganic hybrid Ruddlesden-Popper perovskites (RPPs) quantum well materials [50]. The latter demonstrated superior environmental and photo stabilities compared to their 3D counterparts. Zhang et al. [30] were the 1st to demonstrate the application of this type of perovskite semiconductors as a gain medium in a laser device. The authors demonstrated the fabrication onto polydimethylsiloxane (PDMS) of high-density NW laser arrays. The latter RPPs based NWs operated as FP laser cavity systems with very high Q-factors (1800). Key finding was the observation of optical gain as a function of the number of RPPs films employed; with a nonlinear kink in the optical output intensity to be observed for configurations that employed more than five films. The demonstrated array system was fabricated onto the PDMS substrate with the distances between the NWs to be well defined and controlled. This is a very crucial parameter for controlling destructive interreference effects. The lasing was observed at 72.5 μJ/cm^2^ at 548 nm. The demonstrated stability under ambient conditions was impressive for a hybrid system: more than six hours uninterrupted lasing for over 2.1 × 10^7^ excitation cycles.

#### 2.1.2. Whispering Gallery Mode Cavity Architecture

The Whispering Gallery Mode Cavity (WGM) bases its operation principle in total internal reflection (TIR). In WGM cavities the optical modes are guided by the TIR is provided in the circumference of a circular or polygonal degenerate oscillator (the oscillator is shaped around the gain medium); for example, conformal coating of perovskites onto glass microspheres. Planar WGM cavities also have been demonstrated such as perovskite nanoplates. These configurations are less demanded regarding their engineering compared to the FP cavities and usually fabricated using via atomic laser deposition of the material within the structures substrates and cavities. There are very attractive laser sources since are of high demand in silicon photonics. The highest Q-value reported is of the order of 1650 and threshold values as low as 11 μJ/cm^2^ (which is three order of magnitude higher than these ones achieved following FP cavities). It must be mentioned that the Q-factor is time dependent as a function of the oscillating waves decay. The higher threshold values are attributed to less strong optical confinement the oscillating radiation experiences and of higher losses due to absorption from the material the gallery is made within the cavity. Such laser devices have a potential in quantum information technologies.

Microdisc lasers (MDLs) are convenient laser cavity configurations (Figure 3c) since they are characterized by (a) small mode volume; (b) high Q-cavity, that allow them to be easily embedded into nanophotonic circuits. Their operation is based on the whispering gallery mode (WGM) type resonator, where the optical feedback is secured through the total internal reflection (TIR) along the perimeter of the disc. Liao et al. [28] applied the principles of MDLs using as a gain medium hybrid inorganic-organic perovskites (MAPbIBr_3_). The authors showed, a single longitudinal mode operation and tuning of the lasing emission wavelength achieved through the partial replacement of Br with Cl atoms; managing a tuning range from 525 nm to 557 nm (with decreasing x from one to zero in CH_3_NH_3_PbCl_x_Br_3−x_ crystals). However, the PLQE was getting lower as the x increased (e.g., PLQE ~1% at x = 1 whereas PLQE~22% at x = 0). This was attributed to the increased number of traps as the x was approaching the unity value.

The development of nanophotonic devices that will open new avenues for realizing on-chip optical components has been realised. Perovskite semiconductors due to their attractive physical characteristics and especially their direct bandgap and high optical gain, are promising materials for realizing such an optical component. 3D cavity shapes such as cubic corner pyramids due to the internal effective optical coupling/trapping offer, provided by the three reflectors, show the way towards the realization of low threshold laser devices. Mi et al. [51] provided a theoretical work on MAPbBr_3_ cubic corner pyramids (the three facets operate as very efficient reflectors), chemical vapor deposited onto mica substrates, laser-based elements. Lasing from the perovskite pyramids was observed from 80 to 200 K within the threshold range from 92 μJ/cm^2^ to 2.2 mJ/cm^2^, after the pyramids have pumped by femtosecond laser pulses at 400 nm. In comparison to the inorganic semiconductors, a blue shift of the output wavelength was observed as the temperature raised. This tendency was attributed to the thermal expansion of the crystal lattice and thus the increase of the energy bandgap. As in all other cases, the lasing action was accompanied with a substantial spectral reduction of the output: the FWHM decreased from 9.6 nm to 0.56 nm. No lasing has been observed for temperatures higher than 200 K. By capping an Ag layer beneath the mica substrate, the authors managed to reduce the threshold from 92 μJ/cm^2^ to 26 μJ/cm^2^ (@ 80 K) and achieved lasing operation at room temperature lasing operation (with threshold at 75 μJ/cm^2^). The Ag substrate enhanced the optical feedback from below towards backwards to the MAPbBr_3_ nanopyramids and thus room temperature lasing was simulated.

#### 2.1.3. Random Lasing Architecture

Random lasing (RL) exploits the polycrystalline nature of the perovskite semiconductors. Random scattering between the grains of the perovskite material is exploited as optical feedback to sustain lasing. It is the less engineering demanding “optical resonator” since does not require the construction of an external optical resonator but provides the less control regarding the directionality of the output and spectral tuning of the laser output. Moreover, random lasing requests higher optical gain from the active medium. It also suffers from interference effects since the distance between the various emitters cannot be controlled or be adjusted. Due to the output emission with low spatial coherence and laser-like energy conversion efficiency, RLs are attractive devices for energy efficient illumination application and are promising candidates for electrically pumped UV lasers, biosensors and optical information processing

Random lasing scheme is very attractive for its low cost, simple processing schemes and the freedom to be realised without the use of any artificial designed resonant cavity. Random lasing light source with credentials to be infiltrated into biological tissues and to serve in pinpoint detection, has been rarely explored yet. Random lasing exploits the scattering effects to provide the necessary optical feedback and thus the optical gain. The emission of the induced coherent radiation occurs in all the directions and thus this is another concrete difference with the formally defined directional laser operation. In perovskite polycrystalline materials the scattering is offered by the various interfaces formed between the different grains formed during the crystallization of the material. Shi et al. [29] managed to demonstrate random lasing using CH_3_NH_3_PbI_3_ (450 nm in thickness) films as the gain medium, at room temperature (see Figure 3d). The gain medium was optically pumped using ns laser pulses at 355 nm. The laser threshold was measured at 102 μJ/cm^2^. Coherent light emission was detected at RT and at 775 nm with a FWHM of 0.4 nm; the random lasing operation was confirmed by (a) the random spacing of the observed emission longitudinal modes; (b) the random change of the emission peaks with the optical pumping intensity; and (c) the multi-directionality of the output emission

An important figure of merit of any type of laser device is its laser threshold; it should be as low as possible in order to maximize the conversion of the pumping energy to output power. Kao et al. [52] discovered that the relative concentration of the PbI_2_ played a major role in the determination of the excitonic and lasing performance of the MAPbI_3_ materials. Specifically, the increase of the concentration of the PbI_2_ up to 30 wt.% resulted in the lowering of the lasing threshold, mainly due to (a) increased volume of the gain media; (b) the enhancement of the generated scattering effects (that provide the necessary optical feedback to observe stimulated emission—random lasing) between the grain boundaries; and (c) the higher exciton binding energy (as thermal PL measurements showed). On the other hand, the increase of the PbI_2_ concentration beyond an optimum concentration, due to the incomplete induced crystallization of the perovskite material, resulted in higher lasing threshold values. The random lasing, with threshold value at 230 μJ/cm^2^, was observed at 745 nm. The lasing emission wavelength could be tuned from 745 to 800 nm by modulating the PbI_2_ concentration between 30 and 40 wt.%.

The primary methods to tune a hybrid or an inorganic perovskite laser output are (a) through halide stoichiometry control (Figure 2); (b) through the organic cation substance; and (c) through the concentration of lead iodide precursor. All these methods were applied during the fabrication of the semiconductor. Zhang et al. [53] proposed something innovative since the proposed technique is a post-synthetic. The authors proposed the exposure of CH_3_NH_3_PbBr_3_ microplates with chlorine in inductively coupled plasma, which enabled them to tune the emission wavelength by 50 nm. More importantly, the lasing threshold and the crystallization phase did not change after the application of the proposed process compared to the pristine sample. The increase of the process time from 0 to 60 s resulted in a blue shift of the absorption and the emission wavelengths. A linear relationship between these two experimental factors, with a slope of −0.75 nm/s and total tunability of 50 nm was experimentally extracted. The achieved wavelength tuning was attributed to the modifications of the mixture of halide concentration during the interaction of the hybrid perovskite with the chlorine ions. The lasing of the pristine hybrid perovskite was observed at 555 nm; the lasing threshold was measured at 3.4 μJ/cm^2^ with a FWHM beyond lasing threshold of 0.33 nm (Q-factor of 1667). The lasing wavelength could be tuned with the processing time; tuning from 555 nm (at 0 s) to 506 nm (at 60 s) was observed.

Random Lasing (RL) has only been demonstrated in halide perovskites only via linear one pulse (1P) and nonlinear two laser pulses absorption processes. In contrast, the frequency up conversion of three pulses absorption and resultant RL is still lacking, in spite of its great importance in high—order nonlinear optical applications. Moreover, the 1P pumped cases employ ultra violet (UV) light that is destructive for the samples under investigation. Additionally, due to the high absorption coefficient, the halide perovskites exhibited in this spectral region limit UV laser pulse penetration depth which is a disadvantage especially in biomedical imaging and sensing applications. In contrast, the non-linear up conversion of two or more photons demonstrate a lot of merits, such as: (a) large penetration depth; (b) high spatial resolution; and (c) small photo damage and photo bleaching. Moreover, the multi-photon pumped process has fascinating characteristics such as repression of unwanted absorption losses. In the study by Weng et al. [54], high quality CH_3_NH_3_PbBr_3_ perovskite thin films (thickness of ~4.3 μm and grain sizes ranging from several hundred nanometres to a few microns), were synthesized through a solution based one spin coating method, demonstrating a frequency up converted RL via direct nonlinear 3P absorption process. Apart of the aforementioned advantages compared to the 1P absorption induced lasing, the 3P frequency up conversion films demonstrated lower threshold compare to the systems that have been pumped with 1P. The absorption of the tested films was set at 550 nm (indicating the films’ energy band gap @ 2.27 eV). The films excitation using femtosecond (fs) pulses at 1300 nm resulted in an incoherent RL emission signal at 545 nm, a clear indication of the non-linear 3P up conversion absorption. The necessary optical feedback required by lasing, resulted from random scattering effects provided by the polycrystalline grain boundaries. In addition to a steep increase in the emission intensity at 550 nm, the onset of the observed incoherent RL was accompanied with a simultaneous decrease of the spectral full half width maximum (FWHM) of the detected optical signal. The pump threshold was measured to be approximately 27 mJ/cm^2^. At the same time, an observed wavelength red shift (of maximum 2.8 nm) in the lasing output, as a result of many body interactions, was observed. The measured pulse width of the 3P pumped films, was measured using Kerr—based TRPL and found equal to 3.1 ps, which was the shortest observed in bromide perovskite-based lasers without any post processing. The production of ps laser pulses is one of the attractions of the perovskite-based laser devices. The ultrafast rise process of the RL pulse has benefited from the high material’s optical gain, while the shortest decay time was attributed to the short lifetime of the photons within the perovskite gain medium.

Safdar et al. [55] were motivated from the challenge to fabricate low cost, solution processed and without the need of careful engineered optical cavities, laser devices. The authors managed to show random lasing (single and dual laser mode operation) from a thin film of solution processed perovskite (CH_3_NH_3_PbI_3_), deposited onto a nonpatterned glass substrate at room temperature. The demonstrated lasing threshold was 10 μJ/cm^2^ and the pumping source was a pulsed frequency doubled YAG laser (pulse length of 400 ps, at 532 nm). The light amplification was optimised by finding the optimum point between intense optical scattering and scattering length. The random lasing was observed when constructive interference of the multi-scattered light was secured. It was essential in the random lasing for the number of scattering events and the gain to interplay correctly (not too much or too low scattering effects). By simply controlling the solution processing (spin coating turns, dip time in solvent) the quality and the thickness of the perovskite film was fully controlled and determined; and moreover, the whole random lasing operation was optimised (especial important the optimum crystallization of the perovskite as this was determined by anti- solvent extraction time). The laser waveguide was consisted of air-CH_3_NH_3_PbI_3_-glass and its length was of the order of 50 nm. The threshold value increased as the time of exposure of non-encapsulated perovskite films in air and light increased (e.g., the laser output deceased to 80% of its initial value after its illumination with 105 pulses).

All Organic-Inorganic Perovskite Based Laser Device Characteristics are summarized in Table 1.

### 2.2. Inorganic Perovskite Based Laser Devices

Inorganic based perovskites demonstrate higher environmental and photostability stability compared to the hybrid perovskite systems. On the other hand, the inorganic perovskite semiconductors have higher densities of defect states that reduce their photoluminescence performance (due to the non-radiative recombination). Another big advantage they offer is that very controlled configurations such as quantum dots and nanocrystals can be realized [56]. Inorganic perovskite QDs and nanocrystals are characterized by attractive features are the (a) size dependent spectral emission; an increase of the energy bandgap is observed and thus a blue shifting of the PL outputs with the shrinking of the crystal size (b) demonstrated enhanced photoluminescence due to the well-known size dependent quantum confinement. The inorganic based perovskite QDs are emerged as competitive materials in lighting applications to the currently under industrialization CdSe or InP QDs. Their simpler construction processes, the narrower achieved FWHMs and lower fabrication costs are decisive parameters for their dominant lasing materials. As in the case of hybrid perovskite laser systems, all the types of laser cavity configurations have been demonstrated. Each one of them, as has been already analysed, demonstrates its own advantages. All Inorganic Perovskite Based Laser Device Characteristics are summarized in Table 2.

#### 2.2.1. Fabry-Perot Laser Cavities

Quantum dots (QDs) lasers are size tuneable, low threshold laser devices with a great potential to be employed as single photon sources (optical qubits) with applications in quantum computing. The optical properties of the organic-inorganic halide perovskites (CH_3_NH_3_PbX_3_, X = I, Br, Cl) have shown their potential as efficient optical gain media. Wang et al. [57] combined the advantages of the QD configuration, the photophysical properties of perovskites (high PLQE) and the superior stability against environmental hazards (moisture and oxygen) of the inorganic semiconductors, in order to demonstrate for the first time, stimulated emission at room temperature from optically pumped caesium lead halide perovskite (CsPbX_3_) QDs (~9 nm in diameter). The lasing threshold was measured at 22 μJ/cm^2^ (with PLQE of 85%) which was at that period, one order of magnitude lower than the corresponding values of the CdS QDs. The lasing was not only exhibited at room temperature but it was also sustainable under atmospheric environment exposure; even after three weeks exposure to the air, the QDs exhibited the same lasing threshold value. Moreover the CsPbX_3_ QDs film demonstrated excellent photostability, under the operational conditions; the CsPbX_3_ QDs output was almost at the same lasing intensity level (~90% of the initial output), under optical pumping of 57 μJ/cm^2^, for about 4.5 h The tunability of the lasing wavelength was achieved through the tuning of (a) the size of the QDs (e.g., the 5.5 nm QDs emitted light at 502 nm); and (b) the halide constitution of the perovskite (scan across the whole visible spectrum).

The work of Yakunin et al. [58] shed light to the photophysics of inorganic metal halides (CsPbX_3_) in the form of uniform, size tuneable nanocrystals. Low threshold and tuneable lasing were demonstrated mainly due to the excellent crystal quality & high PLQE (~70–90%) and the compositional tuning of the halide components respectively. The authors demonstrated lasing operation under (a) WGM cavity configuration; (b) FP cavity; and (c) free of optical oscillator configurations (random lasing).

The hybrid perovskite NWs beyond their advantages demonstrate material instabilities: they are sensitive to environmental hazards such as moisture and oxygen. On the other hand, the inorganic Cs based perovskites offered an alternative that show superior stability under environmental operational conditions. Fu et al. [59] employed CsPbX_3_ (X = I, Br or Cl) and MAPb(BrCl)_3_ alloyed composites NWs as an active layer to demonstrate stable lasing operation under ambient conditions. The authors tested various inorganic perovskite films under continuous optically pumped conditions; the inorganic perovskite semiconductor media retained their lasing output constant for more than 7.2 × 10^9^ pumping laser pulses (eight hours of continuous operation). This stability performance was substantially more robust than their organic-inorganic counterparts. The emission spectrum of MAPb(BrCl)_3_ was tuned, as in other similar cases, through the entire visible spectrum, by controlling the stoichiometry of the alloyed perovskite material.

Perovskite semiconductor nanowire lasers have already been demonstrated as a promising approach with well-defined one-dimensional geometry towards on chip integration laser sources. Wang et al. [60] work was motivated by the challenge to understand deeper the operational principles of these configurations: Understanding light matter interactions for both fundamental and practical reasons is critical in designing low power consumption nanoscale light sources. The CsPbX_3_ nanowires are vapour deposited on sapphire deposition layer whereas their average length was of the order of 10–20 μm and their diameter of 200–500 nm. The Rabi splitting (2 g) provided a measure of the photon—exciton coupling; it can be expressed as $g\propto \sqrt{\frac{f}{V}}$ where f is the oscillator strength and V is the mode volume. Since nanowire is an 1D construction and due to strong confinement, the V takes small values and thus the Rabi splitting is expected to be strong. Moreover, due to high excitonic binding energy the oscillatory strength f is high and this allows the stronger exciton—photon coupling within CsPbX_3_ nanowires. Authors’ findings showed that it is these polaritons that were the origin of the demonstrating lasing under CW or pulsed optical pumping. Lasing emission under pulsed optical pumping (using fs laser pulses at 400 nm for the CsPbCl_3_ and 470 nm for the CsPbBr_3_ and CsPbI_3_) was observed only by the two ends of the nanowires as a result of the strong coupling of the spontaneous emission with the FP resonator modes of the nanowire cavity. The stimulated emission appears for threshold pumping values of ~4 μJ/cm^2^ in the case of CsPbBr_3_. The FWHM of the dominating lasing mode at 535 nm, is of the order of ~0.20 nm. The achieved CsPbBr_3_ nanowire lasers have a very high degree (98%) of linear polarization. Moreover, the emitted laser light could be tuned from 425 nm to 722 nm by varying the nanowires chemical stoichiometry.

The superior operational stability (60 min under operational conditions at ambient atmosphere), the tunability achieved through the halide stoichiometry and the excellent optical confinement of the 1D nature NW configurations, using single crystal inorganic perovskite semiconductors were demonstrated from Park et al. [61]. The low lasing (@ 530 nm) threshold approximately at 3 μJ/cm^2^ was secured by the excellent crystallinity of the spin coated CsPbX_3_ NWs (~10 μm long and 300 nm in diameter) and the high oscillatory strength of the 1D character of NWs. The suburb properties of the 1D optical confinement in combination with the excellent crystallinity were reflected on the high Q-value of 1300 of the optical resonators formed between the NW end facets. The experimental measurement of the non-single valued FSR of the laser oscillating modes suggested that the traveling waves within a NW were governed by the exciton-photon dispersion curve (polariton dispersion curve) where the experienced group refractive indexes are 6–7 fold increased; as a result, the oscillating modes appeared closer (smaller FSR) than the classical model predicted.

**Figure 5:**
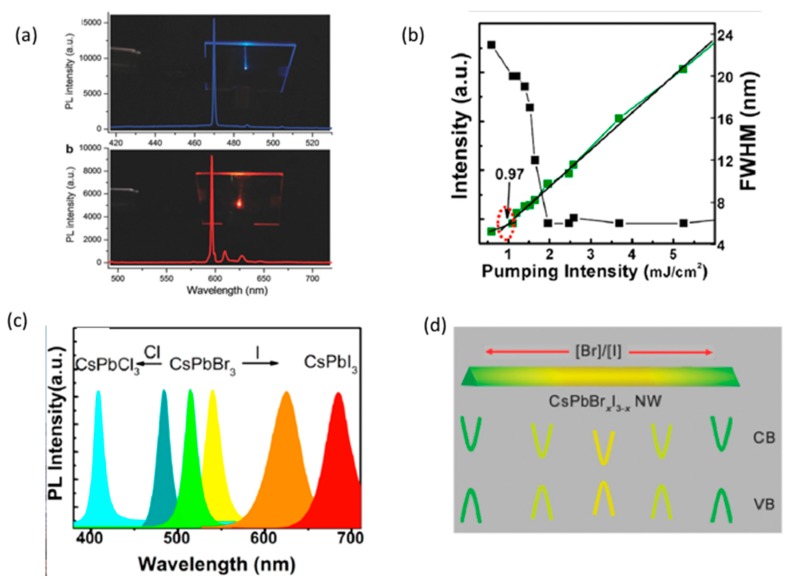
(**a**) Single Mode Laser Operation at blue and red colour from CsPb(Br/Cl)_3_ and CsPb(I/Br)_3_ IPNCs under pump intensity of 38.2 and 30.5 µJ cm^−2^, respectively. Reproduced with the permission from Wang et al. [63], Advanced Functional Materials; published by Wiley-VCH, 2017. (**b**) The transition from the spontaneous emission to stimulated emission is accompanied by a simultaneous non-linear increase of the Intensity above threshold and at the same time a shrieking of the emission spectral linewidth; in this figure the lasing behaviour of CsPbBr_3_ NC film. Reproduced with permission from Tang et al. [70], Nano Energy; published by Elsevier, 2016. (**c**) Tunability of the emission PL light achieved through different composition. Reproduced with permission from Tang et al. [70], Nano Energy; published by Elsevier, 2016. (**d**) Dual Wavelength Lasing through structure of composition-graded CsPbBr_x_I_3−x_ NW and the graded bandgap. Reproduced with permission from Huang et al. [62], Advanced Materials; published by Willey-VCH, 2018.

An innovative synthesis of a graded composed CsPbBr_x_I_3−x_ NWs, that allowed the dual wavelength laser emission, due to the increased Br/I ratio from the centre to the ends, was demonstrated by Huang et al. [62]. A tuneable wavelength emission was exhibited with a blue shifted emission from the centre towards the NWs edges (as a result of the wider bandgap semiconductors at the end while narrower are formed at the centre) (Figure 5d). The synthesis of such construction was challenging because the inorganic perovskite semiconductors due to their soft lattice and rapid anion exchange reaction favours homogeneous alloys to be formed rather than nonhomogeneous ones. The authors exploited (a) the desynchronized deposition of Caesium lead halides; and (b) the temperature-controlled anion exchange reaction to manage the construction of the graded composited CsPbBr_x_I_3−x_ NWs. The varied content along the NW was confirmed from XRD spectra. The CsPbBr_x_I_3−x_ NWs were pumped using fs laser pulses (150 fs, 1 kHz) at 400 nm. Dual colour lasing, with a wavelength separation of 35 nm (from 521 nm to 556 nm) was observed as the micro-photoluminescence (μPL) measurements demonstrated. Both wavelengths appeared into the detected spectrum when the pumping fluence was above 51 μJ/cm^2^. The authors discovered that the emission wavelengths originated from the centre and the ends of the NWs were dependent on (a) the dimensions (length and width of the NWs; following the tendency for further blue shifted as these dimensions are increased); and (b) the fabrication time which determined the ratio of Br/I; as a result of this parameter the nanowire started from deposition of rich in iodide, CsPbBr_x_I_3−x_ (emission above 580 nm) and gradually replaced by a Br rich sample (emission below 580 nm).

Wang et al. [63] demonstrated, for the first time, a vertical cavity emission surface laser (VCESL)—an inorganic perovskite (CsPbX_3_) nanocrystal. This work was exceptional since for the first time a single device demonstrated (a) low threshold; (b) operational stability; (c) single mode operation; and (d) output directionality (one of the most important advantages of a laser device). The laser emission demonstrated very high directionality, with the far field divergence angle to be ~3.6°. The short cavity length secured the single longitudinal mode laser operation at 504 nm (linewidth of ~0.6 nm). The demonstrated VCESL was optically pumped using fs laser pulses at 400 nm. The exhibited system was very stable, with its output retaining 80% of its peak power for more than one hour. Beyond its operational characteristics, these devices presented high flexibility, since this particular VCESL can be versatilely engineered by independent adjustments of the optical resonator and the composition of the perovskite semiconductor crystals (mainly due to the solution processability of the active layer). A step towards the demonstration of a CW perovskite laser device was the demonstration from the authors of quasi steady state lasing. This was achieved using 5 ns and at 400 nm laser pulses. The lasing threshold was at 900 μJ/cm^2^ but as was said this was a big step towards CW optical and electrical pumped perovskite semiconductor lasers. These are important results, since they indicate the roadmap for the development of low-cost laser devices for the future.

The employment of 3D structures, such as micro cubes, can offer lasing operation in three dimensions and therefore the laser action is less sensitive to the excitation direction. Another attractive feature of the perovskite material (independent of their structural set up i.e., 1D or 2D or 3D) is the presence of nonlinear properties that allow the multi-photon pumping. The multi-photon pumping results in lower material damage and the resulted up conversion generates wavelength with (a) deeper penetration depth; and (b) higher spatial resolution. However and regardless of their superb optical properties, the hybrid perovskite semiconductors are very susceptible to hydrolysis and this hinders their commercialization. This stability limitation is addressed with the use of inorganic perovskite materials such as Caesium lead halide perovskite, CsPbX_3_. Hu et al. [64] exploited the advantages of the micro-tubes 3D character and the ability to multi-photon optical pumping them. The micro-cubes side dimensions (505 nm) & facets, operated as Fabry-Perot cavities that resulted optical cavities of very high Q values (~1100) and lasing operation of low threshold at room temperature. Moreover, for two photon optical pumping at 800 nm (using a Ti:sapphire, fs laser system) the threshold was approximately found at 439 μJ/cm^2^. Above this pumping value, the presence of lasing operation was accompanied with a substantial decrease of the spectral width of the output (from 22 nm down to 0.46 nm) and an abrupt increase on the output. The authors, through TRPL spectroscopy measurement, showed that above threshold the carriers’ recombination (excitons recombination) was much faster than below threshold pumping operation due to the lasing effect (1.13 ns from 4 ns). The stability of the inorganic perovskite microcubes was tested under storage and illumination conditions. All the tests occurred in room temperature and under humidity conditions (~35–40%). The storage stability tests showed that after several months storage the micro-cubes retained their structure (XRD measurement was used for this purpose). The photo stability tests acquired PL spectra for several months without showing any significant degradation. A constant ASE signal was measured for over ten hours indicating a significant operating lifetime under ambient conditions.

Liu et al. [65] reported the first laser device that was smaller in all dimensions than the emitted wavelength. Their research was motivated by (a) the requirement to develop optical components with the three of their dimensions in the nanoscale, for applications on chip photonic information processing systems. This is a challenge since the miniaturization of the cavity length and the extremely high requirement of optical gain to overcome optical losses are difficult to realize; and (b) to address the temperature instability of the CsPbBr_3_ semiconductor laser medium. The authors reported the building of a CsPbBr_3_ perovskite nanocuboid laser, with sides’ length of an average of 400 nm, that operated under single longitudinal mode (emitted from a single nanocuboid perovskite source) operation at room temperature. The single mode operation was secured by the short FP distance between the facets of the nanocuboid that made the Free Spectral Range (FSR) longer than the gain bandwidth of the active medium. The flat and smooth end facets of the nanocuboids and the high gain of the material (502 cm^−1^) helped to achieve low threshold lasing operation. The reported threshold lasing values, were 40.2 and 374 μJ/cm^2^ for one (pumped by a fs laser source at 400 nm, emitting laser light at 539 nm with a linewidth at 0.26 nm and a Q-factor of 2075) and two photon (pumped by an fs at 800 nm laser source, emission at 539 nm with a linewidth of 0,29 nm and Q equal to 1859) excitation. The demonstrated quality factor of the nanocuboid laser systems was reported as high as 2075. Moreover, the authors exhibited a quiet stable laser device with temperature insensitive (22 ps—pulse width) lasing operation for temperatures between 180 and 380 K. The operational stability is one important issue for the practical exploitation of these nanolaser systems. The long-term stability under ambient conditions (23 °C, 40% relative humidity) was demonstrated. The presented system showed an excellent photo- and thermal stability without demonstrating any mode hopping after 160 min of uninterrupted excitation. The thermal stability of these systems was evaluated for the temperature window between 77 and 300 K by examining their PL signal after two photons excitation. The impressive thermal stability of these systems is attributed to the strong exciton binding energy of the CsPbBr_3_ nanocuboids. This exhibited stability, between 180 and 380 K, makes them a very promising material for light emitting devices for which temperature arises during their operation and any shift in the emitting wavelength is not desired at all.

CsPbX_3_ based thin films are simpler to be fabricated than nanowires or nanocrystals, however, they contain a lot of pinholes that seriously degrade their lasing performance. This is the reason that despite of their fabrication simplicity, these systems demonstrate much lower quantum efficiency than the corresponding nanocrystals. Li et al. [66] realised that by mixing ZnO nanoparticles (smaller diameter than 35 nm) into the CsPbX_3_ precursor’s solution addressed the aforementioned drawback; the resulted thin film (CsPbBr_3_:ZnO, thickness of 210 nm) synthesized using the one step method, exhibited no pinholes or voids and as a result, its luminesce performance improved compared to the one of the reference device. Criteria of the improved photoluminescence performance was the lower ASE (from 0.292 mJ/cm^2^ to 0.207 mJ/cm^2^ for the one photon pumping and from 0.679 to 0.569 mJ/cm^2^ for the two photon pumping) and lower lasing threshold for one (400 nm) and two photon (800 nm) fs pumping (50 fs, 1 kHz repetition rate, at 800 nm); This was attributed mainly to morphological reasons: (a) Better crystallization (more intense and sharper XRD peaks) that is caused due to the improved nucleation the ZnO nanoparticles induced; (b) Smaller grain crystals (SEM measurements showed decrease of the grain size from 0.43 μm to 0.24 μm) that lead to shorter exciton diffusion lengths and thus less probability for exciton dissociation; (c) less surface roughness (AFM measurements showed a smoother films, the roughness decreased from 36.36 nm to 26.51 nm) that allow the more efficient pumping photons to be trapped within the material; and (d) shorter optical loops the signal is doing between the perovskite crystals. The PL emission spectra of the pristine and the CsPbBr_3_:ZnO thin films is identical (@ 523.5 nm) but with the PLQY of the ZnO NPs devices to be higher: from 12% to 21%. The ASE signal from CsPbBr_3_:ZnO thin films is approximately 4.25-fold higher than that of the pristine thin films.

Evans et al. [67] were the first ones that demonstrated CW lasing from CsPbBr_3_ NWs at 77 K. This is a step towards the realization of electrically pumped perovskite semiconductor lasers. NWs due to their geometry, emit a low laser mode volume that encourages a lot the light matter interaction between the generated excitons and the pumping photons (plasmons). In this regime there is no need of population inversion for stimulated light to be emitted. This tackles the obstacles of high temperature instabilities the perovskite suffers under intense optical pumping to create population inversion. To be better studied, the perovskite nanostructures were optically pumped at room temperature using fs laser pulses at 454 nm. One of the most striking observations in this work was the increase of the FSR as the temperature of the perovskite samples was elevated from 77 K to 294 K. Such a behaviour contradicted the function of the NW as a photonic based FP cavity. The authors suggested that the CW lasing observed at very low temperatures was ascribed to generated polaritons which revealed the strong matter interaction in this material. This work introduced the low power inorganic perovskite polaritonic based lasers.

The majority of the work done until today regarding the hybrid organic-inorganic perovskite lasers is characterised from an output emission in the NIR part of the spectrum. There is a lack of demonstrators in visible of low cost and stable perovskite lasers. Low cost, stable under ambient conditions, solution processed laser devices in visible are very important for applications including displays, high-density data storage and readout and underwater communications. This gap will be filled by the inorganic perovskite materials. The latter materials compared to the hybrid ones, demonstrate some superior properties such as (a) higher current densities; (b) reduced heating effects; (c) superior thermal and environmental stabilities. Gong et al. [68] exhibited the unique merits of thin film (80 nm), solution processed inorganic perovskite semiconductors (CsPbBrI_2_-PEO) as a gain medium in DFB laser configuration. Polyethylene oxide (PEO) was added into the precursor solutions to improve the film quality, resulting in an enhanced PLQY values. The CsPbBrI_2_-PEO systems demonstrated single mode operation, high gain coefficient of 161.1 cm^−1^ that with the combination of the provided optical feedback from the constructed nanoprinted DFB resonator (with the Bragg grating period to be 360 nm), resulted in low lasing threshold values of 33 μJ/cm^2^, of linear polarised (TE—optical polarization parallel in the grooves of the grating) emissions at 654 nm, with a linewidth of 4.9 nm. The samples were pumped by a 355 nm 90 pico-second laser.

#### 2.2.2. Whispering Gallery Modes

The continuous evolvement and the upcoming commercialization of the integrated onto chip photonics circuits request the fabrication of low cost nanolaser sources. The work from He et al. [69] proposed a one step, room temperature, facile and of low-cost fabrication process of well-spaced aligned micro-disk (MDLs) Caesium lead halide-based laser arrays. The proposed technique permitted the researchers of this work to control the size and the spacing of the fabricated onto quartz CsPbX_3_ MDLs. Key element of the proposed technique was the employment of a PDMS cylindrical hole template that confined the fabrication of the CsPbX_3_ in well predefined positions and sizes. The CsPbX_3_ MDLs were optically pumped by a 400 nm, 150 fs laser pulses at 1 kHz. The lasing threshold was similar for all the constructed MDLs, measured around 10.3 μJ/cm^2^ at 425 nm, an attractive feature since it permitted the simultaneously operation of all the photonic elements onto the quartz substrate. The stochiometric tunability was also demonstrated and allowed the spectral tuning of MDLs output (from 416 nm to 530 nm). The fabrication of different wavelength output MDLs onto the same substrate was also exhibited; a configuration especially important for multi-coloured laser displays, laser lighting and sensing.

#### 2.2.3. Random Lasing Cavities

Tang et al. [70] combined the advantages of the inorganic perovskite semiconductors and the facile operational principles of the random lasing; they managed to demonstrate a very low cost and of low threshold (0.97 mJ/cm^2^) (Figure 5b) optically pumped miniature laser source (QDs of diameter of 9 nm). Random lasing operation, under optical excitation using a 100 fs laser source at 800 nm, was observed. The emission wavelength depended on the halide element selected; lasing peaks at 427 (CsPbCl_3_), 527 and 539 nm were observed (Figure 5c).

## 3. Conclusions

The field of perovskite based nanolasers since 2014 has advanced the leveraging of various gain media and laser cavity configurations. The optical confinement secured by the low dimensional perovskite material configuration, in combination with the superb intrinsic properties (e.g., solution processability, high absorption and emission coefficients in visible, excellent and defect free crystallinity, smooth end crystal facets, high carrier injection ratio, long bipolar diffusion lengths, low non radiative losses) and the advancements in device engineering, have replicated their potential into solar cells to that of laser devices. Moreover, the formation of natural optical cavities exploiting the smooth facets of the perovskite grains is another attractive feature of these semiconductor materials towards the realization of simpler, low cost and solution processed nanolaser systems. On the other hand the optical confinement the nanoperovskite configurations offer is counter balanced by the higher existence of surface/interface induced trap states between the crystal grains that diminish their emission performance. So, an optimization in engineering of these devices is still a challenge.

The current optically pumped laser systems are the beginning of the trip. The aforementioned perovskite semiconductor properties and laser device performance indicate that the main challenge and ultimate target to realize electrically pumped perovskite lasers will be a reality soon. However, other milestones should be reached before perovskite-based laser devices evolve beyond research labs and attract market stakeholders. First of all, the low exciton binding energy—approximately few meVs—is a limiting factor to realize efficient electrical pumped perovskite-based laser elements. This challenge it seems that can be addressed with the engineering of QDs and nanocrystals as gain media in the laser devices. The introduction of lead (Pb) free, efficient light emitters will be also a breakthrough (for environmental purposes) allowing the easier commercialization of these devices. The exploitation of the high quantum yield, the spectral tunability and purity, the solution processability of the perovskite materials, will be fully exploited only if the operational stability under ambient conditions, especially in the case of organic-inorganic perovskite semiconductors, is improved and the respective devices’ operational lifetime extended.

For enhanced environmental stability maybe 2D perovskites semiconductors are the solution. The introduction of two dimensional Ruddlesden-Popper Perovskites is an excellent selection for environmental stable lasing perovskite applications. In general, 2D perovskite are more stable than 3D but the photovoltaic performance of the 2D perovskite is still quite low, which is a challenge that should be overcome for the laser devices as well. An alternative is the selection of inorganic perovskite-based laser system that have reported with superior environmental stabilities compared to the hybrid systems

Another stability challenge apart of the environmental hazards (that can be addressed using high quality encapsulation coatings) the perovskite-based lasers face are the high temperatures the intense optical pumping or the high charge injection and the laser emission generate. Increasing the optical pump laser’s pulse duration is essential to produce enough population inversion and sustain lasing. However, the high generated temperatures have a negative impact on the photostability of these materials. Graphene based materials can be applied in perovskite-based lasers, within the active layer or as a thin interlayer and due to their high thermal conductivity can operate as heat sinkers and extend the lifetime and the lasing time of these devices. This will be a very big thrust for this laser technology. Moreover, the more efficient temperature handling, will allow the optical pumping of these devices with ns laser sources reducing the operational costs and at the same time doing a step towards the continuous wave laser and electrically driven lasing. The pairing of perovskite active layers with materials such as graphene that convey heat more efficient, will allow the razing of the ablation threshold of the perovskite film higher than its lasing threshold. This will be a major step towards the realization of continuous wave lasing operation from these solution processed materials. However more work should be invested into the understanding of the photo-degradation mechanisms of these materials in order to treat them more effectively.

A step towards the realization of more stable laser gain media could be the demonstration of triple cation perovskite laser device that exhibit better stability as materials. Moreover, these materials provide a control of the achieved crystal quality as a function of the lead deficiency as well. The coupling of these materials with grating structures and the demonstration of single mode laser operation is expected to open the field of continuously tuneable perovskite laser devices and the latter devices application in high demanded laser based spectroscopic applications.

Another major challenge is the coupling of the coherent laser light outside of the gain medium. This is mainly secured with the coupling of the active layer with interlayers of higher refractive index. A major step forward for the realization of electrically driven lasing devices will be the adoption of transparent conductive electrodes for example, reduced graphene oxide or other metal oxides instead of metal electrodes that will facilitate the extraction of the generated laser light.

In conclusion, perovskite semiconductors are characterized by all the characteristics an ideal laser gain medium should have; slow radiative non-radiative pathways, large mobilities, high carrier densities. It seems that the in depth understanding of this star material will further facilitate its application as an electrically drivel laser source. It is highly expected that the research in perovskite-based laser systems will be intensified in the near future showing the potential of these materials beyond solar cells. Perovskites are in the spotlight in the field of new emerging optical materials for lasing.

## Figures and Tables

**Table 1:** Organic-Inorganic Perovskite Based Laser Device Characteristics; NA denotes for the Non-Available Information.

Material	Nanostructure Type/Cavity Architecture	Laser Threshold (μJ/cm^2^)	Emission Linewidth (nm)	Emission Wavelength (nm)	Q-Factor	Gain Coefficient (cm^−1^)	Tunability (nm)	Pumping Source	Reference (Year)
MAPbI_3−x_Cl_x_	Thin Film/Vertical Cavity (FP)	0.2 μJ/pulse	N/A	775	NA	NA	NA	0.4 ns @ 532 nm	Deschler et al. (2014) [26]
MAPbI_3_	NWs/Fabry-Perot Cavity	220 nJ/cm^2^	0.22	787	3600	NA	400–800	100 fs @ 402 nm	Zhu et al. (2015) [27]
MAPbIBr_3_	MDLs/WGM	3.6	1.1	557.5	430	NA	525–557	150 fs @ 400 nm	Liao et al. (2015) [28]
MAPbI_3_	Thin Film/Random lasing	102	0.4	775	NA	NA	NA	1 ns @ 355 nm	Shi et al. (2016) [29]
Ruddlesden—Popper Perovskite	2D NWs/Fabry-Perot Cavity	72.5	0.3	548	1800	87.3	NA	@ ~487 nm ^1^	Zhang et al. (2018) [30]
MAPbI_3_	Thin Films/DFB systems	120 kW/cm^2^	0.2	786.5	NA	NA	781–794	1 ns @ 532 nm	Brenner et al. (2016) [31]
MAPbI_3_	Photonic Crystal/Fabry-Perot Cavity	68.5	0.24	788	NA	NA	768–794	270 ps @ 532 nm	Chen et al. (2016) [32]
MAPbBr_3_	MWs/Fabry-Perot Cavity	674	0.8	546	650	NA	NA	100 fs @ 800 nm	Gu et al. (2016) [33]
MAPbBr_3_	Micro-rods/graphene/Fabry-Perot Cavity	48	N/A	551.8	N/A	NA	NA	N/A	Zhang et al. (2016) [34]
MAPbBr_3_	Microplates/WGM	3.4	0.33	555	1667	NA	555–506	NA	Zhang et al. (2016) [34]
MAPbI_3_	Platelets/WGM	11	N/A	780	1200	NA	NA	120 fs @ 400 nm	Liu et al. (2016) [35]
(FA, MA)PbI_3_ and (FA, MA)Pb(I, Br)_3_	NWs/Fabry-Perot Cavities	6.2	0.24	560 (FAPbBr_3_)	2300	NA	500–790	150 fs @ 400 nm	Fu et al. (2016) [36]
CH_3_NH_3 × 3_	Plasmonic NWs/Fabry-Perot Cavities	13.5	5	785	151	NA	767–796	120 fs @ 400 nm	Yu et al. (2016) [37]
MAPbI_3_ & MAPbBr_3_	Thin Films/Fabry-Perot Cavities	NA	NA	NA	NA	NA	NA	130 fs or 4 ns ^2^	Sarritzu et al. (2016) [38]
MAPbBr_3_	Nanoribbons/Fabry-Perot Cavities	4.2	0.7	553	NA	NA	NA	100 fs @ 400 nm	Sun et al. (2017) [39]
MAPbX_3_	NW arrays/Fabry-Perot Cavities	18.3	0.9	543.1	500	NA	NA	150 fs @ 400 nm	Liu et al. (2017) [40]
CH_3_NH_3_PbBr_3_	Photonic Crystal/DFB (FP)	1.6	0.15	545	NA	NA	NA	0.5 ns @ 532nm	Shunemann et al. (2017) [41]
CH(NH_2_)_2_Pb(I_1−x_Br_x_)_3_	Thin Films/DFB (FP)	3.5	NA	630	NA	NA	550–820	400 ps @ 532 nm	Cha et al. (2017) [42]
CH_3_NH_3_PbBr_3_	Microsheet/Fabry-Perot Cavities	0.251 mW	0.41	554	1352	NA	NA	150 fs @ 325 nm	Chen et al. (2017) [43]
CH_3_NH_3_PbI_3_	Thin Films/DFB (FB)	17 kW/cm^2^	NA	785	NA	NA	NA	920 ns @ 445 nm	Jia et al. (2017) [44]
CH_3_NH_3_PbI_3_	Thin Film/Ring Cavity Laser (FB)	19.6	NA	791.5	650	NA	Possible	120 fs @ 645nm	Cegielski et al. (2017) [45]
CH_3_NH_3_PbBr_3_	Microplates and Microrods/WGM	130	0.3	547	1800	NA	NA	100 fs @ 1240 nm	Gao et al. (2017) [46]
CH_3_NH_3_PbX_3_	Microplates/WGM	30	NA	770	3200	NA	550–770	100 fs @ 400 nm	Huang et al. (2018) [47]
CH_3_NH_3_PbBr_3_	Thin Film/DFB (FB)	3.4	0.14	555	NA	NA	543–557	0.3 ns @ 532 nm	Pourdavoud et al. (2018) [48]
CH_3_NH_3_PbI_3_	Thin Film/VCSEL(FB)	12.9	0.76	782	312	NA	750–760	150 fs @ 633 nm	Wang et al. (2018) [49]
CH_3_NH_3_PbBr_3_	Cube Corner Pyramids/Fabry-Perot Cavities	75	0.56	530	NA	NA	NA	400 nm	Mi et al. (2018) [51]
MAPbI_3_	Thin Films/Random lasing	230	3	745	NA	NA	745–800 ^3^	0.5 ns @ 355 nm	Kao et al. (2016) [52]
CH_3_NH_3_PbBr_3_	Thin Film/Random Lasing	27 mJ/cm^2^	NA	545	NA	NA	NA	35 fs @ 1300 nm	Weng et al. (2018) [54]
CH_3_NH_3_PbI_3_	Thin Film/Random Lasing	10	45	NA	NA	70.1	NA	400 ps @ 532 nm	Safdar et al. (2018) [55]

^1^ Optically pumped @ ~487 nm for five films of RPPs; ^2^ 130 fs (CW), 4 ns (quasi CW); ^3^ with varying the concentration of PbI_2_.

**Table 2:** Inorganic Perovskite Based Laser Device Characteristics; NA denotes for the Non-Available Information.

Material	Nanostructure Type/Cavity Architecture	Laser Threshold (μJ/cm^2^)	Emission Linewidth (nm)	Emission Wavelength (nm)	Q-Factor	Gain Coefficient cm^−1^	Tunability (nm)	Pumping Source	Reference (Year)
CsPbX_3_	QDs/WGM	22	NA	524.5	NA	98	Possible	100 fs @ 400 nm	Wang et al. (2015) [57]
CsPbX_3_	Nanocrystals/FP Cavities and WGM cavities	5	0.15	530	10^9^	450	440–700	100 fs @ 400 nm	Yakunin et al. (2015) [58]
CsPbX_3_ & MAPb(BrCl)_3_	NWs/FP Cavities	6.2	0.26	538	2069	NA	420–710	150 fs @ 402 nm	Fu et al. (2016) [59]
CsPbX_3_	NWs/FP Cavities	4	0.2	535	2256	NA	425–722	100 fs @ 400 nm	Wang et al. (2018) [60]
CsPbX_3_	NWs/FP Cavities	3	0.3	530 (of CsPbBr_3_)	1300	NA	NA	100 fs @ 355 nm	Park et al. (2016) [61]
CsPbBr_x_I_3−x_	NWs/FP Cavities	51	0.3	534	NA	NA	521–566	150 fs @ 400 nm	Huang et al. (2018) [62]
CsPbX_3_	Thin Films/VCESL (FP)	9	0.6	504	NA	NA	400–700	100 fs @ 400 nm	Wang et al. (2017) [63]
CsPbX_3_	Micro-cubes/FP Cavities	439	0.46	533	1100	NA	420–630	35 fs @800 nm	Hu et al. (2017) [64]
CsPbX_3_	Nanocuboids/FP Cavities	40.2	0.29	539	2075	502	NA	100 fs @ 400 nm	Liu et al. (2018) [65]
CsPbX_3_ with ZnO NPs	Thin Films/FP Cavities	569	NA	530	NA	NA	NA	50 fs @ 800 nm	Li et al. (2017) [66]
CsPbBr_3_	NWs/FP Cavities	6 kW/cm^2^	NA	532	2300	NA	NA	1 W, CW laser diode at 450 nm	Evans et al. (2018) [67]
CsPbBrI_2_-PEO	Thin Films/DFB (FB)	33	4.9	654	NA	161.1	NA	90 ps @ 355 nm	Gong et al. (2017) [68]
CsPbX_3_	Micro-disc Arrays/WGM	10.3	NA	425	530	NA	416–530	150 fs @ 400 nm	He et al. (2017) [69]
CsPbBr_3_	QDs/random lasing	0.97	6	539	90	NA	400–700	100 fs @ 400 nm	Tang et al. (2016) [70]

FP, Fabry-Perot.
